# The clinical and economic consequences of practice style variations in common surgical interventions: A protocol for systematic review

**DOI:** 10.1097/MD.0000000000012439

**Published:** 2018-10-19

**Authors:** Mojtaba Nouhi, Mohamad Hadian, Alireza Olyaeemanesh

**Affiliations:** a School of Health Management and Information Sciences, Iran University of Medical Sciences; b National Institute of Health Research; c Health Equity Research Center, Tehran University of Medical Sciences, Tehran, Iran.

**Keywords:** practice style variations, surgical intervention, systematic review, unwarranted variation

## Abstract

**Background:**

Surgical intervention is one of the common therapeutic interventions applied to a vast class of diseases. Unwarranted variation in practice style in different locations is considered as practice style variations (PSVs), which cause undesirable effects on patient health status and economic consequences. The magnitude of the variations in surgical interventions and its effects on clinical outcomes of patients and also utilization of resources have been investigated in recent years. But the findings show considerable heterogeneities in magnitude and consequences. We develop a protocol to systematically review the current literature of PSV to explain the magnitude of PSV and its clinical and economic consequences.

**Method:**

This systematic review will include observational and experimental studies to investigate magnitude and consequences of PSV in common surgical interventions, cardiovascular disease, urological, and ophthalmological diseases. Source of information is scientific databases, theses, clinical trials registrations website, and grey literature. A comprehensive electronic search will be conducted through PubMed, Web of Science, EBSCO, EMBASE, and Scopus databases. Studies are assessed systematically by 2 investigators. Methodological quality of the included studies is evaluated by the STROBE and CONSORT checklists. In case of data availability, we will pool findings of included studies by meta-analysis techniques in the CMA software. Subgroup analyses are based on the type of the interventions and selected diseases.

**Results:**

This study has ethical approval from ethical committee of Iran University of Medical Sciences, ethic code: IR.IUMS.REC1395.9221504203. The results will be published in a peer-reviewed journal.

**Conclusion:**

A systematic review is considered as an appropriate scientific method for reaching a consensus on magnitude as well as consequences of PSV. Results of this study will help clinical experts to attain more knowledge about PSV and encourage them to use some tools such as clinical guidelines and shared decision making to alleviate its consequences.

## 1 Introduction

Clinical practice variation is recognized as diversity in utilization of treatment and diagnostic services across different geographic locations. Several studies have shown that there is a considerable variation in providing diagnostic and treatment services.^[1–5]^ On the basis of the findings of Dartmouth Atlas of health care, receiving heart-vascular, oncologic, and orthopedic services varied between 3 to 10 times in different geographic locations of the USA.^[6,7]^

A national survey in Australia indicated that women living in rural and suburban areas had experienced hysterectomy for abnormal uterine bleeding 5 times more than those living in urban areas.^[8]^ Such findings are observed in other countries, including Spain, Canada, and the UK.^[9–11]^ Furthermore, studies have shown that the probability of receiving different surgeries by patients is equally related to both the patient's clinical and health state, and his/her residing area.^[12]^

Notice, variation in health service provision is not unfavorable by itself. If all variations in surgical interventions provision are undesirable, diminishing it would not seem much difficult.^[13]^ In fact, different factors from different levels come together and cause variation in surgical interventions provision. In the personal level, an amount of variation in service provision is related to the patient's clinical features. As the patients are not located equally in different areas, an amount of variations can be derived from patient's preferences, that is, the patient cooperates in the clinical decision making process on whether doing or refusing surgical interventions.^[14,15]^

The evidence emphasize variations in surgical intervention also can be caused by differences in the ratio of the number of physicians to a location of people, types, and levels of service provision in a hospital or to the available beds in hospitals of a location in comparison to another one. However, existence of some degree of variations in service provision can be related to diffuse technological innovations.^[16,17]^

But some studies discussing the factors affecting such variations in service provision have pointed out that service providing variation cannot be utterly and solely impressed by patients’ related variables,^[18]^ also there is a considerable number of patients receiving surgical interventions less or higher than their need,^[19]^ which affects patients’ health status and decreases the service provision quality.^[20–22]^ This part of variations is the unwarranted variations, which could be stem in style of practitioners and called practice style variation (PSV).

PSV incurs undesirable effects on patients’ health by providing services less or more than patients’ needs leads to inequity in patients’ access to surgical interventions, and an inappropriate distribution leads to important and unfavorable clinical outcomes and financial consequences in the society. Moving further away from an optimum level of services, which should be provided for patients leads to inefficiency in using the resources of the health care system.^[23,24]^

It can be expected that decreasing the unwarranted variation in surgical interventions can be adopted as a way of improving efficiency. In recent years, a variety of studies have investigated the magnitude and the consequences of PSV surgical interventions. Considering the literature review, no systematic review was found investigating the clinical and economic effects of variation in surgical interventions. This study aims to systematically review the clinical and economic consequences of variation in surgical intervention of 3 common diseases.

The research questions of this study are as follows:

(1) What degree of variations in surgical interventions is considered as PSV in surgical interventions?
(2) What is the extent of undesirable clinical consequences caused by PSV in surgical interventions?
(3) What is the extent of the economic consequences caused by PSV in surgical interventions?

## 2 Method

### 2.1 Eligibility criteria

#### 2.1.1 Study characteristics

We include observational (case–control, case report, case series) and interventional (experimental studies, clinical controlled trial, randomized controlled trial) studies to investigate the clinical and economic effects caused by PSV in surgical interventions. The Preferred Reporting Items for Systematic reviews and Meta-Analyses for Protocols 2015 (PRISMA-P 2015) have been used for preparing the protocol of this systematic review.^[25]^

In this study, the clinical consequences mean a change in mortality rate of patients, incidence of physical and mobility disabilities, and emergence of morbidities such as reduction in quality of life, pain, or anxiety.

Economic consequences of PSV mean a shift in resources utilization, length of stay in hospital, out of pocket payment of patients, and finally on a macro level, it means welfare loss. This outcome can be either reported directly in form of monetary values, or indirectly through converting the economic effects to monetary values. PRISMA guidelines are used for formulating the results and providing a final report on them.^[26]^

#### 2.1.2 Type of participants

Patients with cardiovascular, urological, and ophthalmological diseases who underwent surgical interventions are the participants of this systematic review. No age, race, or gender limitations are applied. Setting of studies are not restricted to specific countries.

The primary outcomes are the degree of PSV in surgical interventions and the level of mortality, disability rates caused by PSV. Secondary outcomes of interests are morbidity in terms of quality of life and pain and anxiety of patients due to PSV. We also consider differences in resources utilization, differences in length of stay of patients in hospital, and amount of their payment and welfare loss caused by PSV.

#### 2.1.3 Setting and timeframe

In this systematic review, all relevant studies, theses, and reports in the time span of 1880 to 2018 are considered.

#### 2.1.4 Report characteristics

No language restrictions are considered for retrieving potentially eligible studies with abstract in English language.

#### 2.1.5 Information source

We use electronic database, website of registry interventional studies, and grey literature as source of information to explore potentially eligible studies. We systematically search PubMed, Web of science, Scopus, EMBASE, and EBSCO databases using appropriate terms. References of marker studies are also searched as a complementary action for finding further studies. Furthermore, international databases such as World Health Organization (WHO), The European Public Health Association (EUPHA), and websites of the atlas of variation in health services in countries such as Australia, UK, and USA are manually reviewed. For assuring from the universality of the searching, theses in the field of investigating PSV found in the ProQuest website are also entered. For recovering the abstracts of the scientific conferences, Scopus and Web of Science databases and other related websites are also searched. We electronically contact with experienced authors and scholars in field of PSV to find any unpublished details in articles, or if there are any unpublished studies, they could be included as well. Moreover, the list of articles published in journals related to the field of PSV such as journal of evaluation in clinical practice are reviewed.

### 2.2 Searching strategy

For designing the search strategy, Cochran guidelines will be used.^[27]^ A vast searching strategy will be formed in 3 compartments with consideration of the research questions. For searching the studies related to the first research question stating the extent of variation in performing clinical surgeries, the 2 compartments 1 and 2 are integrated using AND. For recovering the researches related to the second and third research questions expressing the clinical and economic effects of variation in performing surgeries, the compartments 1 and 3 are integrated using AND as well. In the first step, a searching strategy using words such as MESH and other related words will be formulated in PubMed for recovering articles. Then, this searching strategy will be modified and utilized for other databases. The initial searching strategy is as follows:

(1) variation pattern[Title/Abstract] OR interventional variation [Title/Abstract] OR surgical variation[Title/Abstract] OR practice variation[Title/Abstract] OR practice style[Title/Abstract] OR unexplained variation[Title/Abstract] OR medical variation[Title/Abstract] OR surgical variation[Title/Abstract] OR health professional practice style[Title/Abstract] OR unwarranted practice variation[Title/Abstract] OR warranted variation OR clinical variation OR observation [Title/Abstract]
(2) Rate of variation [Title/Abstract] OR percentage of variation [Title/Abstract] OR intra-class variation [Title/Abstract], proportional variation OR small area variation [Title/Abstract] OR atlas of variation [Title/Abstract] OR geographic distribution [Title/Abstract] OR geographic comparison [Title/Abstract]
(3) Consequences [Title/Abstract] OR impact [Title/Abstract] OR utilization [Title/Abstract] OR adverse effect [Title/Abstract] OR Iatrogenic impacts [Title/Abstract] OR patient satisfaction [Title/Abstract] OR mortality [Title/Abstract] OR morbidity [Title/Abstract] OR disability [Title/Abstract] OR quality of life [Title/Abstract] OR [Title/Abstract] pain [Title/Abstract] OR welfare loss [Title/Abstract]
(4) 1 AND 2 (to find relevant studies regarding question 1)
(5) 1 AND 3 (to find relevant studies regarding question 2 and 3)

### 2.3 Study records

#### 2.3.1 The selection process of studies

Studies are reviewed independently by 2 reviewers skilled in the research subject. In the first step, the title and abstract of the recovered studies are reviewed. After that, the full text of the remaining studies is carefully investigated for checking if they match the input and output measures of the present study. The remaining researches are entered into the final analysis. Each reviewer organizes the results of using the study selection process in a file. The studies, which are validated by both reviewers, enter the analysis step; the studies that are not validated by the reviewers based on the research measures exit the analysis process. Those studies about which the reviewers do not agree upon are assessed by a third reviewer. This process continued until consensus is reached.

#### 2.3.2 Studies quality

Quality assessment questionnaires are adopted in order to investigate the methodological quality of studies. On the basis of the design of the qualified studies, their related expert questionnaires are used. The Strobe questionnaires, which are developed on specific design distinctions, are considered as an appropriate tool for assessing the quality of studies. This questionnaire includes several questions related to designing various studies and biases allowing us to compare studies based on their methodological quality. The quality assessment results of the analysis input studies are demonstrated in a separate table.

#### 2.3.3 Extraction and management of evidences

The information related to each of the input studies is extracted in a pre-designed form. The information not only includes initial data such as publication year, the design method of studies, authors’ names, and the studies country, but it also includes specific information such as the study population, statistical method, the studies perspective, findings, and the evidences analysis methods as well. This form will be filled for each include studies by 2 reviewers independently. In case of disagreement between the reviewers on extracting data, the subject will be discussed with third reviewers to reach a consensus. Considering the information of each study, a special code is dedicated to each of them, so that further analysis would become more feasible.

#### 2.3.4 Evidences analysis

The findings of all studies are collected in detailed tables. These studies are compared in terms of their initial features, their reported variation extent, clinical, and economic effects. The publication time process of studies and the methods applied for detecting unwarranted variation are analyzed; the publication time process and for homogeneous studies, findings are collected quantitatively and through meta-analysis techniques. A suitable statistical method will be selected with regard to data type. Results are reported in terms of relative risk with a confidence distance of 95%. The homogeneity level between studies will be evaluated through the *l*^2^ test. In case the amount of *l*^2^ is more than 50%, it shows high inhomogeneity between studies. Studies’ findings are collected using the fixed-effect and random effect methods. *P* = .05 was statistically significant. Funnel plot is used for investigating publication bias. In case the evidences are sufficient, subgroup analysis will be performed based upon the type of surgery and disease.

## 3 Discussion

Variation in common surgical interventions is one of the challenges and issues of providing care services are also accompanied with clinical effects for patients, and economic effects for the health system.^[28]^ This protocol systematically reviews the evidences in the field of variation level in performing clinical intervention surgeries, evaluating the clinical and economic consequences led by such variation.^[29]^ Evidences systematic review is a useful scientific technique for reaching the answers to complicated questions. The present studies are the first systematic reviews investigating these questions. By publishing the findings from this study, the clinical experts’ awareness of therapeutic services variation and their luck in adopting appropriate actions such as following clinical guidelines and shared decision making can be increased for managing the variations.

## Author contributions

**Conceptualization:** Mohamad Hadian

**Formal Analysis:** Mojtaba Nouhi, Mohamad Hadian, Alireza Olyaeemanesh

**Funding Acquisition:** Mohamad Hadian

**Methodology:** Mojtaba Nouhi, Alireza Olyaeemanesh

**Project administration:** Mojtaba Nouhi

**Supervision:** Mohamad Hadian

**Writing original draft:** Mojtaba Nouhi

**Writing – review & editing:** Mojtaba Nouhi, Alireza Olyaeemanesh, Mojamad Hadian

Mojtaba nouhi orcid: 0000-0002-4466-8403
